# Left Bundle Pacing for Left Bundle Branch Block and Intermittent Third-Degree Atrioventricular Block in a *MYH7* Mutation-Related Hypertrophic Cardiomyopathy With Restrictive Phenotype in a Child

**DOI:** 10.3389/fped.2020.00312

**Published:** 2020-06-16

**Authors:** Luyan Zhang, Xueying Cheng, Jinlong Chen, Ming Zhou, Tianwei Qian, Zhongman Zhang, Jie Yin, Han Zhang, Genyin Dai, Yuming Qin, Shiwei Yang

**Affiliations:** Department of Cardiology, Children's Hospital of Nanjing Medical University, Nanjing, China

**Keywords:** hypertrophic cardiomyopathy, restrictive phenotype, conduction block, left bundle pacing, *MYH7*, genetics

## Abstract

Hypertrophic cardiomyopathy (HCM) is a group of myocardial diseases defined by cardiac hypertrophy which cannot be explained by secondary causes with a non-dilated left ventricle and preserved or increased ejection fraction. Sometimes it can be combined with restrictive cardiomyopathy. Here we describe a very rare case of a 12-year-old girl with non-obstructive hypertrophic cardiomyopathy accompanied by restrictive phenotype, complete left bundle branch block and intermittent third-degree atrioventricular block, who presented with recurrent syncope. Her father was also found to have hypertrophic cardiomyopathy and treated with implantable cardioverter defibrillator for ventricular tachycardia. Her younger brother is currently asymptomatic but echocardiogram showed hypertrophic cardiomyopathy. Genetic analysis identified a heterozygous missense mutation (c.2155C>T, p.R719W) of *MYH7* in the proband girl, her father and her brother. The girl was treated with left bundle pacing and recovered well. The case we present further demonstrates the feasibility of left bundle pacing in children.

## Introduction

Hypertrophic cardiomyopathy (HCM) is a group of myocardial diseases characterized by left ventricular hypertrophy unexplained by secondary causes and a non-dilated left ventricle with preserved or increased ejection fraction, which can be divided into obstructive HCM and non-obstructive HCM (1, 2). It has been estimated that prevalence of HCM is 1/500 in the general adult population and 0.47/100,000 in children (3, 4). Usually, cardiac hypertrophy is asymmetrical with interventricular septum hypertrophy. Children with HCM can present with syncope, dyspnea, exercise intolerance, peripheral edema and chest pain. Non-sustained ventricular tachycardia and atrial fibrillation are common in HCM while heart block is rare (1). Compared to HCM, restrictive cardiomyopathy (RCM) is a rare form of heart muscle disease with an even smaller incidence of 0.03–0.04/100,000 in children (5, 6). It is known that RCM is characterized by a stiffened ventricle with biatrial enlargement, normal left ventricular wall thickness and atrioventricular valves (5). However, a third of patients with RCM have both ventricular hypertrophy and bilateral enlargement, displaying a mixed phenotype with HCM (6, 7).

Here we report a rare familial hypertrophic cardiomyopathy. The proband was a 12-year-old girl who presented with recurrent syncope and was found to have non-obstructive HCM accompanied by restrictive phenotype, complete left bundle branch block (CLBBB) and intermittent third-degree atrioventricular block (III AVB). Her father and younger brother also had hypertrophic cardiomyopathy. Whole exome sequencing identified a heterozygous missense mutation (c.2155C>T, p.R719W) of *MYH7* in the proband as well as her father and younger brother. Considering the life-threatening conduction block, the proband received left bundle pacing (LBP) and no syncope has reoccurred during more than 1-year follow-up.

### Clinical Presentation

**Figure 1:**
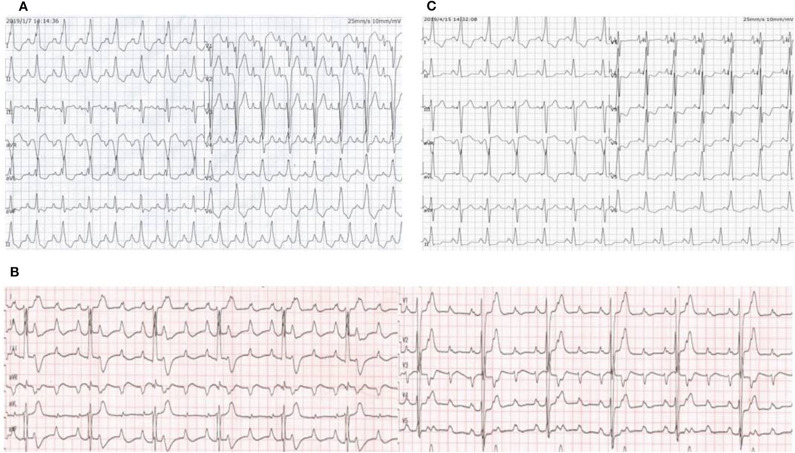
**(A)** ECG on the day of admission showed a regular sinus rhythm, complete left bundle branch block with a QRS complex of 175 ms, first-degree atrioventricular block and bilateral atrial enlargement. **(B)** ECG when the proband experienced syncopal attack again showed third-degree atrioventricular block with a ventricular rate of 40 bpm. **(C)** ECG after LBP showed a narrowing of the QRS and complete right bundle branch block.

**Figure 2:**
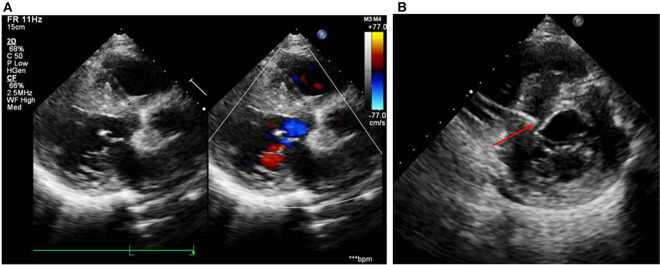
**(A)** Echocardiogram on the day of admission showed damaged systolic function, interventricular septum predominantly thickened (23 mm), biatrial enlargement (LA: 44 mm, RA: 40 mm), posterior wall of left ventricle slightly thickened (10 mm), and moderate mitral regurgitation. **(B)** Echocardiogram during the operation showed the right ventricular pacing electrode located at the upper interventricular septum with a depth of 14.5 mm and a distance of about 3 mm from the left ventricular endocardium.

A 12-year-old girl was referred to the Department of Cardiology for recurrent syncope on January 7, 2019. The girl stated that she felt dizzy and chest discomfort when she was walking, then she fell down with amaurosis fugax. The condition relieved in a few seconds. She once experienced the episode of syncope 3 years ago, but no one paid attention. Her vital signs were as follows: heart rate of 102/min; respiratory rate of 25/min; blood pressure of 127/74 mmHg. Auscultation revealed powerful heart sound without cardiac murmur. The size of liver and spleen were normal. No edema was observed throughout the body. The electrocardiogram (ECG) showed a regular sinus rhythm, complete left bundle branch block with a QRS complex of 175 ms, first-degree atrioventricular block and bilateral atrial enlargement (see Figure 1A). The Holter monitor recorded sinus rhythm, biatrial overload, prolonged PR interval, left ventricular high voltage, complete left bundle branch block, and wide ST-T changes. The echocardiogram showed interventricular septum predominantly thickened (maximum: 23 mm), biatrial enlargement (LA: 44 mm, RA: 40 mm), posterior wall of left ventricle slightly thickened (10 mm), moderate mitral valve regurgitation (see Figure 2A), damaged systolic and diastolic function with left ventricular ejection fraction of 51.8%. Magnetic resonance imaging (MRI) demonstrated biatrial enlargement, hypertrophy in left ventricular wall and septum with a maximal thickness measured at 24 mm and no evidence of pericardial abnormality. Both echocardiogram and MRI showed no obstruction in the left ventricular outflow tract. Etiologic investigations revealed normal plasma amino acids, and urine organic acids. Results of the major laboratory tests were within normal range except for a markedly elevated plasma level of B-type Natriuretic Peptide (BNP) 1,165 pg/ml (upper limit of normal, 100 pg/ml), reflecting the impaired left ventricular function.

**Figure 3:**
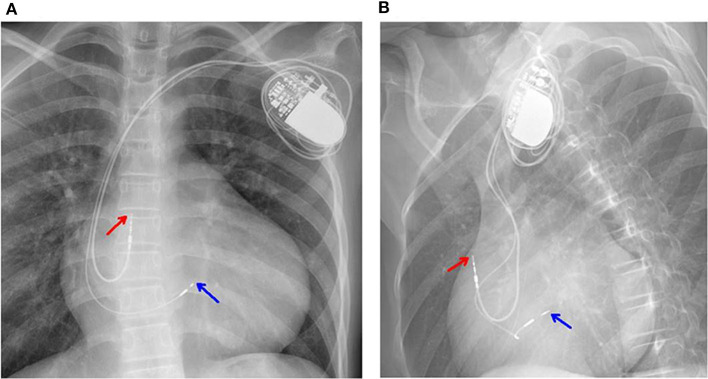
**(A,B)** Chest X-rays showed the 5,076 pacing lead located in the anterior wall of the right atrium (red arrow). The 3,830 electrode passed through the right ventricular septum and reached the sub-endocardium of the left ventricle (blue arrow).

After the exclusion of pericardial abnormalities, combined with the obvious enlargement of bilateral atria and decreased diastolic function in the girl, we made a diagnosis of non-obstructive HCM in combination with restrictive phenotype and complete left bundle branch block. However, it was unable to explain manifestation of recurrent syncope in the proband. On the third day, the proband experienced syncopal attack again with heart rate dropping to 40/min and blood pressure to 65/35 mmHg. ECG showed III AVB (see Figure 1B), which may account for the patient's recurrent syncope. Medical treatment was not effective. Considering the existence of CLBBB and intermittent III AVB with high risk of sudden cardiac death, cardiac pacing was deemed necessary for this girl. As a novel pacing strategy, LBP was taken into account. We applied a Medtronic dual-chamber pacemaker in DDDR mode. The 5,076 pacing lead was placed on the anterior wall of the right atrium while the 3,830 pacing lead passed through the right ventricular septum and reached the sub-endocardium of the left ventricle side (see Figure 3). The lead stopped when ECG QRS configuration was transformed from a CLBBB pattern to right bundle branch block (RBBB) and pacing-to-QRS interval suddenly shortened, which suggest pacing of the left bundle branch. Echocardiography showed it was located at the upper ventricular septum, with a depth of about 14.5 mm and a distance of 3 mm from the left ventricular endocardium (see Figure 2B). Finally, a significantly narrow QRS complex was achieved and CLBBB was reversed with threshold of 1V at 0.4 ms and sensing of 4 mV. No vascular injury, ventricular septal defect, tricuspid valve injury and other complications occurred.

The proband girl further took metoprolol because of sinus tachycardia after implantation and diuretics to lower cardiac preload. In 1 week, echocardiographic measurements were significantly improved (LA: from 44 to 36 mm; RA: from 41 to 39 mm; LVEF: from 51.8 to 59.2%). ECG showed a narrower QRS complex (from 175to 154 ms) and complete right bundle branch block (see Figure 1C). During more than 1-year follow-up, the proband presented no complaints of discomfort, with a heart rate of about 60 bpm at night and 80 bpm in the daytime. Remote monitoring showed the pacemaker working well. In our last follow-up, LVEF was up to 61% and BNP has decreased markedly to 480 pg/ml. However, there was no decrease in interventricular septum thickness.

**Figure 4:**
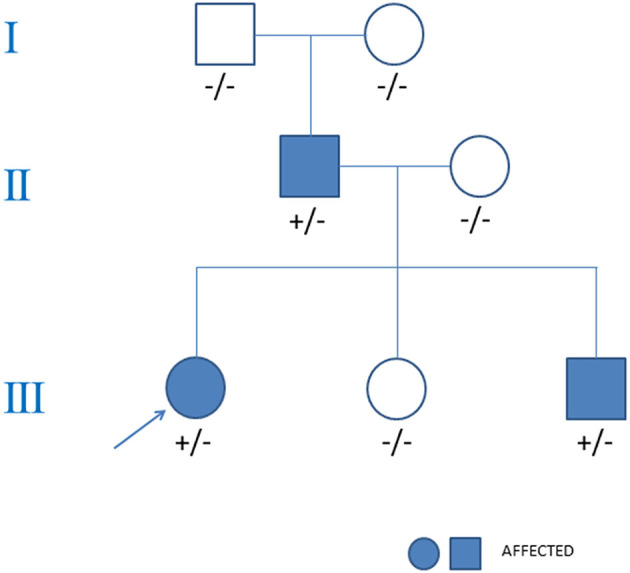
Pedigree analysis of the family in the case. The arrow points out the proband. Circles stood for female. Squares stood for male. The variant *MYH7* c.2155C>T is indicated +/− if heterozygous and −/− if negative.

Familial clinical evaluation showed that both her father and younger brother had HCM (see Figure 4). During the follow-up, her father experienced palpitations and was further treated with implantable cardioverter defibrillator because of ventricular tachycardia. Her younger brother is currently asymptomatic but echocardiogram showed septal hypertrophy. Neither of them had a history of heart block. Her mother, younger sister and her grandparents were all healthy and carry no mutation.

### Genetics Study

Whole exome sequencing identified a heterozygous missense mutation (c.2155C>T, p.R719W) of *MYH7* in the proband girl. This mutation located in exon 19 with a frequency of 0.000032 in the normal population, and GERP++ predicted it was conserved. Bioinformatic analysis based on Polymorphism Phenotyping v2, Sorting Intolerant From Tolerant, and Mutation Taster suggested that the variant is possibly damaging. According to the algorithm ACMG/AMP, this mutation is pathogenic (8). Genetic screening showed that the p.R719W mutation inherited from her father (see Figure 4). Her younger brother also had this mutation, which was absent in her mother, younger sister and her Grandparents. No mutation in arrhythmias related genes such as *SCN5A, KCNH2, KCNQ1, CACNA1C, RYR2*, and *LMNA* was found in the proband or her family members.

## Discussion

HCM is one of the most common phenotypes in pediatric cardiomyopathy (6). Compared to HCM, RCM is a rare form of heart muscle disease with an even smaller incidence of 0.03–0.04 cases per 100,000 children in which one third have a mixed phenotype with hypertrophic cardiomyopathy (HCM) (6). Neither CLBBB nor AVB is common in HCM. In this case, the proband was diagnosed with non-obstructive HCM accompanied by restrictive phenotypes, CLBBB and intermittent III AVB, which is very rare.

Mutations in the sarcomere protein genes have been found to be significant causes of genetic based cardiomyopathies, and the beta-myosin heavy chain (*MYH7*) gene mutations are the most common cause of HCM (9). In this family, we identified the p.R719W mutation of *MYH7* in the proband girl, her father and younger brother, all of whom had the phenotype of HCM, and the healthy mother did not have the mutation, which fully demonstrated that the mutation was co-segregated with the disease. What's more, the p.R719W mutation has been previously reported in several unrelated familial HCMs, with a high incidence of premature death and an average life expectancy (10–12). Therefore, we concluded that the mutation is the leading cause of the disease.

Heart blocks are rare complications of hypertrophic cardiomyopathy, the causes of which are unclear. We firstly suspected that they were caused by mutations in *MYH7* since 31% of HCM patients with *MYH7* mutations showed conduction system disorders such as sinus dysfunction, AVB and bundle branch blocks (13). In this report, there are three subjects with HCM. One of them had ventricular tachycardia attacks and only the proband girl presented CLBBB and III AVB. Other reported carriers of the mutation did not exhibit heart block. However, it should be noted that environmental interactions, age, and sex may influence the penetrance of disease genes, which determine the phenotypes of cardiomyopathy (14). In general, more cases or functional tests are needed to confirm whether the mutation may cause these phenotypes. We also considered the presence of other mutations but genetic screening detected no mutation in arrhythmias related genes such as *SCN5A, KCNH2, KCNQ1, CACNA1C, RYR2*, and *LMNA*. Besides, some reports have pathologically explained heart block in HCM, including interrupted continuity of the conduction system in his bundle, interstitial or myocardial necrosis, small intramural coronary arteries with thickened walls, luminal narrowing in HCM (15–18).

Treatment for this proband girl was very difficult. As she had increased risk of sudden death as a result of CLBBB and III AVB, cardiac resynchronization treatment was an essential option. Traditional pacing modalities are non-physiological, which may limit clinical response. The latest clinical researches demonstrated that left bundle pacing or peri-left bundle pacing (LBP/peri-LBP) using a transventricular septal approach is a physiological pacing method which directly captures the left bundle or ventricular tissue near the left bundle to restore electrical synchronization, which was first reported in 2017 (19). LBP/peri-LBP generates narrow paced QRS duration, fast synchronized left ventricular activation, and correction of left bundle branch block (19, 20). However, LBP/peri-LBP application on children has been rarely reported. Ponnusamy et al. (21) successfully treated a 13-year-old child with LBP for congenital complete heart block in 2019. Huang et al. (22) present a case of a 6-year-old child with III AVB who received a permanent LBP. After all comprehensive consideration, we applied LBP in this proband girl: (A) The position of block was below the His bundle with low ejection fraction. (B) LBP has the advantages of physiological pacing, relatively simple operation, sensing well and lower threshold (23, 24). The implantation process was successful, and postoperative follow-up showed a good recovery. The case we presented further demonstrates the feasibility of LBP/peri-LBP in children.

Although the proband has recovered well after LBP, further follow-up is needed. It is necessary to pay attention to the fact that there are several potential problems such as mechanical damage to the wires caused by ventricular septal contraction. Besides, although left bundle pacing has played a role in improving ventricular function and reducing the size of atria, it is not effective in the treatment of ventricular hypertrophy. There is still possibility of developing ventricular arrhythmias and heart transplantation.

## Data Availability Statement

All datasets generated for this study are included in the article/supplementary material.

## Ethics Statement

Written informed consent was obtained from the participants' legal guardian/next of kin for the publication of any potentially identifiable images or data included in this article.

## Author Contributions

LZ, XC, and JC contributed in case presentation, composing, and editing the manuscript. MZ, TQ, and ZZ cared for the patient and collected sample. HZ, JY, and GD cared for the patient and collected patient's information. YQ and SY assisted in manuscript editing. All authors have read and approved the final manuscript.

## Conflict of Interest

The authors declare that the research was conducted in the absence of any commercial or financial relationships that could be construed as a potential conflict of interest.
